# Impact of COVID-19 Pandemic, Social Vulnerability, and Opioid Overdoses in Chicago

**DOI:** 10.1016/j.focus.2023.100086

**Published:** 2023-02-10

**Authors:** Randall W. Knoebel, Sage J. Kim

**Affiliations:** 1 Department of Pharmacy, The University of Chicago Medicine, Chicago, Ilinois; 2 Department of Medicine, The University of Chicago, Chicago, Ilinois; 3 School of Public Health, University of Illinois Chicago, Chicago, Ilinois

**Keywords:** Social Vulnerability Index, opioid overdose deaths, neighborhood socioeconomic disadvantage

## Abstract

**Introduction:**

This research was undertaken to examine the individual and neighborhood drivers that contributed to increases in opioid overdose deaths during the COVID-19 pandemic.

**Methods:**

The incident location and Centers for Disease Control and Prevention Social Vulnerability Index (along with the individual indicators) were then geocoded to 1 of the 77 Chicago Community Areas. Changes in opioid overdose death rates were calculated and compared for each Chicago Community Area using linear regression between 2019 and 2020.

**Results:**

Opioid overdose deaths increased by 45% from 2019 to 2020. Chicago Community Areas in the highest 25th percentile of social vulnerability before the pandemic had a 2.8 times higher rate of opioid overdose deaths than Chicago Community Areas in the lowest 25th percentile. The increase in opioid overdose death rate observed from 2019 to 2020 was 10.2 times higher in the most socially vulnerable Chicago Community Areas than in the least vulnerable communities. Chicago Community Areas with the highest degree of social vulnerability had a higher baseline and disproportionate relative increase in opioid overdose death rate compared with the least vulnerable Chicago Community Areas.

**Conclusions:**

COVID-19 has revealed the urgent need for policies that better support the social and economic security of disadvantaged communities, particularly for residents who use opioids.

## HIGHLIGHTS

• Opioid overdose deaths in Chicago increased by 45% from 2019 to 2020.
• The CDC Social Vulnerability Index explained the disproportionate increase in overdose deaths.
• Community household composition and economic status correlated with opioid deaths.

## INTRODUCTION

Even before the pandemic, the third wave of the opioid epidemic has been raging regionally and nationally, driven mainly by illicitly manufactured fentanyl making its way into the illicit drug supply.^1^ Each wave of the opioid crisis has only added to rather than replaced the previous waves, with increases in deaths from prescription opioid overdoses since the 1990s, heroin starting in 2010, and a more recent surge in deaths from illicitly manufactured fentanyl starting in 2014.^2^

The U.S. had over 107,000 overdose deaths, the highest number of overdoses deaths reported in a 12-month period in 2021, representing a nearly 15% increase from 2020.^3^ On March 21, 2020, Illinois enacted a stay-at-home order in response to the coronavirus disease 2019 (COVID-19) pandemic. After that announcement, weekly opioid overdose deaths (OODs) increased by 192%, from a baseline of 22.6 deaths per week in December 2019 to an astounding 43.4 per week during the 11-week stay-at-home order.^1^^,^^4^ These elevated rates of OODs have persisted beyond the initial surge of the COVID-19 pandemic, with similar patterns observed in most states across the country.^1^^,^^3^

Non-Hispanic Black men have disproportionally experienced a higher OOD rate since 2013, coinciding with the start of the third wave in the opioid epidemic.^5^ Specifically, in Cook County, Illinois (the county in which Chicago resides), Blacks account for more than half of all opioid deaths in 2019 while making up <24% of the population.^6^ In addition, similar to other diseases of despair,^7^ opioid-related deaths are associated with socioeconomic marginalization, a set of conditions that contribute to exclusion from social and economic opportunities and create vulnerability.^8^ Examples of socioeconomic marginalization include labor market exclusions; informal or illicit income generation (e.g., theft, drug dealing, street-based work); material insecurity (e.g., housing or food insecurity); inadequate income; incarceration; homelessness and housing instability; social stigma or isolation; and low SES or poverty, all of which contribute to OODs.^8^ Although it is often challenging to isolate root causes, patterns visible between these conditions and opioid overdose rates illustrate how together multiple types of vulnerable social and economic conditions affect health inequalities for people who use opioids.

Social vulnerability is a concept that refers to the potential negative impacts on communities caused by external stresses on human health. The Center for Disease Control and Prevention Social Vulnerability Index (SVI)^9^ is a validated measure of community vulnerability to natural or human-caused stressors. The SVI is derived from 15 U.S. Census Bureau American Community Survey variables, which consist of 4 themes: Theme 1: SES (below poverty, unemployed, per capita income, aged >25 years without a high-school diploma), Theme 2: household composition and disability (aged ≥65 years, aged ≤17 years, older than age 5 years with a disability, single-parent households), Theme 3: minority status and language (minority, speak English less than well), and Theme 4: housing type and transportation (multiunit structures, mobile homes, crowding, no vehicle, group quarters such as work dormitories, skilled nursing facilities, or college dorms). The SVI assigns each tract a percentile rank (0−1, with 1 representing the highest vulnerability).

The COVID-19 pandemic has brought with it anxiety, unprecedented levels of unemployment, social isolation, psychological trauma, and general uncertainty about the future.^10^ For individuals with substance use disorder, a group already carrying a disproportionate burden of psychological trauma and mental health disorders, these societal changes brought on by our response to COVID-19 can create what the New York Times termed a national relapse trigger.^11^^,^^12^ Drugs, alcohol, and opioids are frequently used as a refuge from physical and psychological trauma, concentrated disadvantage, isolation, and hopelessness.^13^ Compounded by the fact that social distancing also increases the risk of overdosing alone,^14^ it creates a complex interplay of risk to an already vulnerable population.

This study will utilize a socioecologic model to answer how changes in opioid-related deaths in Chicago are driven by factors operating at multiple levels. By examining the ecologic factors driving OODs, we will understand the vital role context has on health promotion and allow more tangible targets when engaging with communities.

## METHODS

### Study Sample

To determine whether an association exists between higher SVI and OODs, we conducted a retrospective cross-sectional observational secondary data analysis of measures from Chicago census tracts. This protocol was reviewed and approved by the University of Illinois at Chicago IRB (Protocol Number 2022-0477).

### Measures

We obtained the OOD cases from the Cook County Medical Examiner's Case Archive between January 1, 2019 and December 31, 2020. Cases occurring between January 1, 2019 and December 31, 2019 were grouped as the pre−COVID-19 era, and cases occurring between January 1, 2020 and December 31, 2020 were grouped as the COVID-19 era.

We geocoded the incident location information using the ArcGIS GIS software 10.8 (Esri, Redlands, CA). There are approximately 800 census tracts in the City of Chicago, and census tracts are tied to 77 Chicago Community Areas (CCAs), which are well-defined geographic boundaries. We then appended SVI scores to the census tracts and aggregated them into the 77 CCAs. The 14 SVI indicator variables, 4 themes, and the overall composite scores were used at the Chicago community level. The OOD rate (per 100,000 residents) was calculated at the CCA level.

### Statistical Analysis

We performed statistical analysis using Stata, Version 16 (StataCorp, College Station, TX). Statistical significance was defined as *p*<0.05. For bivariate analyses, we analyzed CCAs’ average SVI scores and individual demographic information between time periods using Kruskal−Wallis, *t*-tests were used for continuous variables, and the chi-square test was used for categorical variables. A hierarchical approach was used for the linear regression, starting broadly at the overall SVI, then social vulnerability subdomains (Themes 1–4), and finally individual indicators of SVI only if the higher level was statistically significant. Because we utilized a prespecified hierarchical approach, no adjustment for multiple hypothesis testing was performed.^15^ Using ArcGIS 10.8 (Esri, Redlands, CA), we developed a choropleth map to show the spatial distribution of SVI and OOD rate changes among the CCAs.

## RESULTS

There were a total of 908 opioid-related deaths in 2019, which increased to 1,319 in 2020, representing a 45% increase in opioid-related deaths. The OOD rate increased from 41.6 per 100,000 residents in 2019 to 60.3 per 100,000 residents in 2020, with the average Chicago Community area observing an increase of 19.4 (SD=33.1) per 100,000 residents. In addition, the number of census tracts with no overdose cases decreased by 71%, from 180 in 2019 to 104 in 2020, indicating an expansion of areas where overdose deaths occurred.

Individual demographics for OODs did not significantly shift from 2019 to 2020 (Table 1), with an average age of 47.6 (SD=12.9) years and 77.5% being of the male sex. Non-Latinx Black residents accounted for most OODs, with 56.1% in 2019 increasing slightly to 57.3% in 2020 (*p*=0.168). Non-Latinx White residents accounted for 30.5% of OODs in 2019, which dropped to 26.5% in 2020. Latinx residents saw the largest absolute increase in deaths from 2019 to 2020, making up 12.4% and 15.2%, respectively (*p*=0.168).

**Table 1:** Demographic Characteristics of Individuals Who Died from Opioid Overdose in 2019 and 2020 in Chicago

Demographic characteristics	2019 (*n*=908)	2020 (*n*=1,319)	*p-*value	Total (*n*=2,227)
Age, mean (SD)	47.5 (12.3)	47.6 (13.2)	0.904	47.6 (12.9)
Sex, %				
Female	24.1	21.3	0.114	22.5
Male	75.9	78.7		77.5
Race, %				
Non-Latinx White	30.5	26.5	0.168	28.1
Non-Latinx Black	56.1	57.3		56.8
Latinx	12.4	15.2		14.1
Non-Latinx Asian/pacific islander	0.6	0.5		0.5
Other/unknown	0.5	0.5		0.5

In the year 2019 before COVID-19, the Chicago Community Areas with the highest social vulnerability had the highest rate of OODs at 80.2 per 100,000 (SD=71.2), which is 2.8 times higher than that of the lowest SVI communities with a baseline death rate of 28.4 per 100,000 (SD=25). Linear regression results showed a statistically significant relationship between SVI and the 2019 OOD rate (*p*=0.002). These 2 explanatory variables combined accounted for 12.3% of the explained variability in the change of OODs.

**Figure 1:**
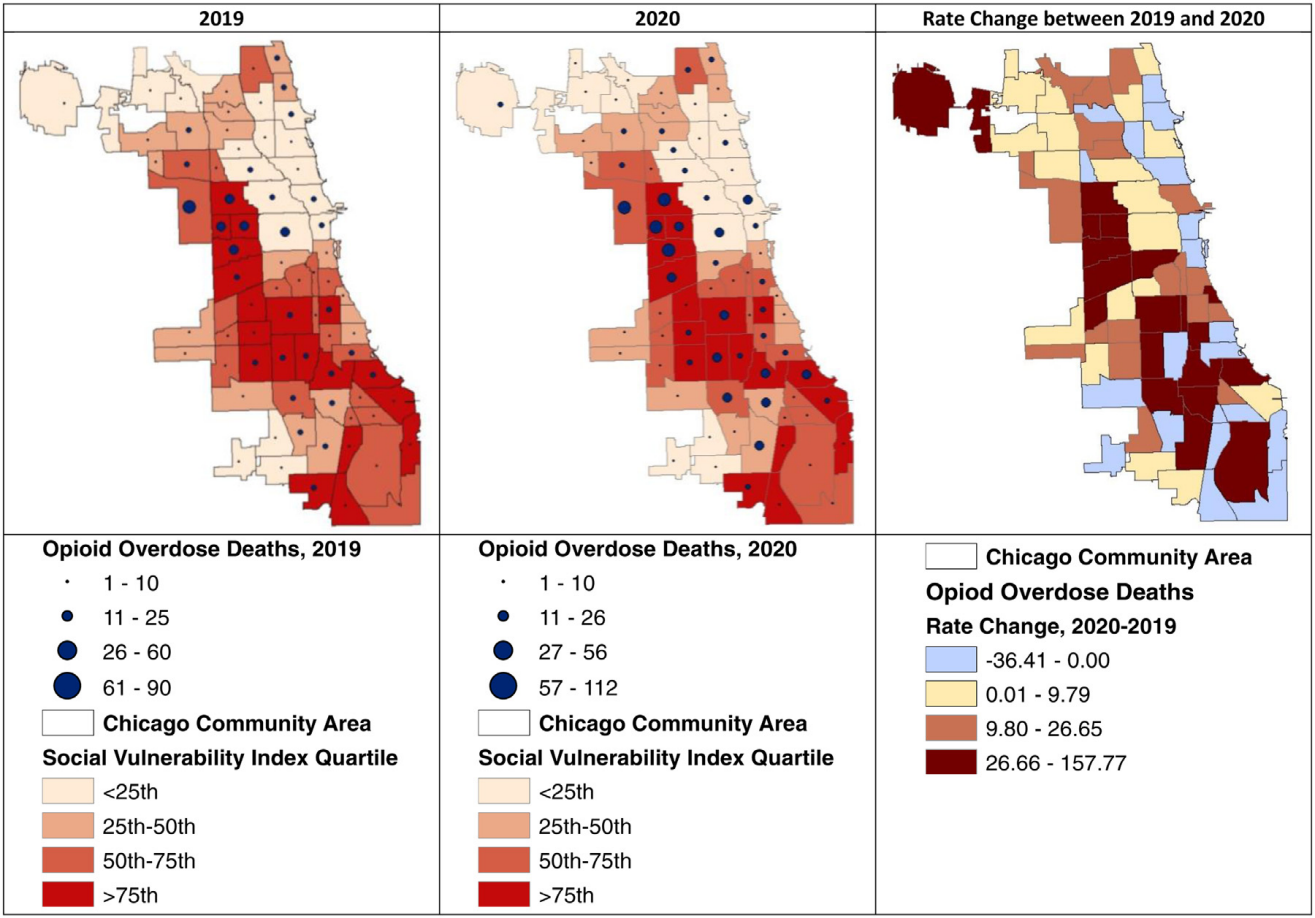
Spatial analysis of overdose death rate per 100.00 residents and SVI. SVI quartile: The Chicago Community Areas with a lighter color represent the less vulnerable area. OOD rate changes: The OOD rate change between 2019 and 2020 was the difference in the rate of OOD in 2020 from the rate in 2019; thus, positive numbers indicate an increase in the OOD rates. OOD, opioid overdose death; SVI, Social Vulnerability Index.

In the year 2020 during COVID-19, the Chicago Community Areas in the highest quartile of social vulnerability had the largest increases in OOD rates by an average of 44.3 per 100,000 (SD=42.3), which is a 10.2-fold higher increase than the lowest SVI communities with an average increase of 4.2 per 100,000 (*p*=0.0001). The relationship between SVI and change in OOD rate was nonlinear. Therefore, a quadratic regression was performed to quantify the relationship between the SVI (measured 0−1) and the corresponding OOD rate change. Results showed that there was a statistically significant relationship between the explanatory variables SVI and SVI^2^ and the response variable change in OOD rate (*p*<0.0001). These 2 explanatory variables combined accounted for 30% of the explained variability in the change of OOD rates. These patterns of overdoses were plotted spatially against the Chicago Community Area SVI, as illustrated in Figure 1, showing the disproportionate impact on the south and west sides of the city.

The lowest SES communities (SVI Indicator 1) had a 50% increase in OOD rate with 80.6 per 100,000 (SD=73.9) in 2019 compared with 121 per 100,000 (SD=110.1) in 2020. Communities in the lowest SES quartile observed an increase of 40.5 per 100,000 (SD=44.2) in OOD rate, which is approximately 6 times higher than the change observed for communities in the highest SES quartiles 6.8 per 100,000 (*p*=0.0013). Communities with a higher percentage of residents (aged ≥65 years, aged ≤17 years, older than age 5 years with a disability, and single-parent households) (SVI Indicator 2) experienced an increase in OOD rate by 36 per 100,000, which is 8.6 times higher risk than that of the communities in the lowest quartile with a 4.2 per 100,000 increase (*p*=0.011). Overall neighborhood indicators for minority status and language (SVI Indicator 3) and housing type and transportation (SVI Indicator 4) were not statistically associated with an increase in OOD rate (Table 2).

**Table 2:** Opioid Overdose Death Rate per 100,000 Residents by Social Vulnerability Index

Social Vulnerability Index (quartile)	Quartile mean (SD)	2019 mean (SD)	2020 mean (SD)	Change mean (SD)	*p-*value	Adj *R*^2^
SVI1: SES					**0.0013**	0.163
Q1	0.27 (0.14)	26.5 (23.6)	33.3 (18.1)	6.8 (21.3)		
Q2	0.77 (0.07)	32.0 (23.3)	36.8 (26.2)	4.8 (23.1)		
Q3	0.87 (0.03)	40.1 (25.2)	62.9 (38.7)	22.8 (23.5)		
Q4	0.98 (0.03)	80.6 (73.9)	121.0 (110.1)	40.5 (44.2)		
SVI2: Household composition and disability					**0.0111**	0.108
Q1	0.25 (0.08)	29.5 (21.5)	33.7 (21.2)	4.2 (26.6)		
Q2	0.56 (0.09)	22.2 (19.8)	32.8 (13.8)	10.6 (16.7)		
Q3	0.79 (0.07)	48.5 (49.5)	71.3 (71.0)	22.8 (27.5)		
Q4	0.97 (0.05)	76.5 (61.7)	112.5 (96.2)	36.0 (44.7)		
SVI3: Minority status and language					0.9299	−0.036
Q1	0.57 (0.13)	39.7 (30.7)	55.3 (36.8)	15.6 (30.5)		
Q2	0.74 (0.05)	72.1 (65.8)	93.9 (108.1)	21.8 (49.3)		
Q3	0.83 (0.03)	42.1 (48.9)	58.8 (67.9)	16.7 (27.3)		
Q4	0.98 (0.04)	27.0 (23.0)	47.6 (38.5)	20.6 (20.8)		
SVI4: Housing type and transportation					0.4372	−0.003
Q1	0.5 (0.1)	25.2 (18.7)	36.6 (25.5)	11.4 (25.1)		
Q2	0.59 (0.03)	39.8 (22.7)	59.6 (32.8)	19.8 (20.8)		
Q3	0.74 (0.05)	64.5 (69.7)	92.6 (109.6)	28.2 (46.0)		
Q4	0.96 (0.07)	49.8 (50.0)	65.1 (66.4)	15.3 (33.3)		
Overall					**0.0001**	0.231
Q1	0.57 (0.15)	28.4 (25.0)	32.5 (19.2)	4.2 (26.2)		
Q2	0.78 (0.06)	35.0 (22.2)	40.7 (29.0)	5.7 (19.3)		
Q3	0.84 (0.02)	30.1 (18.1)	46.9 (24.2)	16.7 (16.5)		
Q4	0.95 (0.03)	80.2 (71.2)	124.6 (103.7)	44.3 (42.3)		

*Note:* Boldface indicates statistical significance (*p*<0.05).

Adj, adjusted; SVI, Social Vulnerability Index.

Individual SVI indicators with the strongest association for increases in OOD rates were communities with a higher proportion of residents below the poverty line (*p*=0.0062) and with a lower median per capita income (*p*=0.0227), a higher proportion of residents without a high-school diploma (*p*=0.0106), and a higher proportion of single female-headed households (*p*=0.0171) (Table 3). Chicago Community Areas located on the south and west side of Chicago carried a disproportionate increase in OOD rates.

**Table 3:** Opioid Overdose Death Rate per 100,000 by Neighborhood Level Indicators

Indicator	Quartile mean (SD)	2019 mean (SD)	2020 mean (SD)	Change mean (SD)	*p-*value	Adj *R*^2^
Below poverty (%)					**0.0062**	0.1234
Q1	8.6 (3.1)	22.3 (21.2)	32.4 (17.0)	10.1 (19.2)		
Q2	16.6 (1.9)	26.1 (18.0)	33.0 (10.1)	6.8 (19.4)		
Q3	24.8 (2.5)	52.2 (45.1)	69.2 (70.9)	17.0 (34.0)		
Q4	37.2 (7.8)	77.9 (64.6)	117.9 (96.1)	40.1 (43.0)		
Unemployed (%)					0.1276	0.0375
Q1	3.8 (1.3)	22.2 (14.6)	31.3 (14.4)	9.1 (19.4)		
Q2	7.8 (1.6)	24.3 (22.3)	36.5 (17.3)	12.2 (19.2)		
Q3	14.6 (2.7)	60.9 (46.3)	81.4 (76.3)	20.5 (41.0)		
Q4	23.1 (4.6)	71.0 (65.4)	103.3 (98.1)	32.3 (41.0)		
Per capita income ($)					**0.0227**	0.0859
Q1	15,217 (1,796)	81.2 (73.7)	116.7 (113.3)	35.6 (50.1)		
Q2	20,150 (1,244)	40.7 (23.8)	65.7 (40.4)	25.0 (25.5)		
Q3	27,693 (2,655)	28.0 (21.1)	38.9 (24.8)	10.9 (21.4)		
Q4	51,204 (16,231)	28.8 (24.9)	35.4 (19.5)	6.6 (20.7)		
No high-school diploma (%)					**0.0106**	0.1092
Q1	5.7 (3.2)	31.3 (25.8)	33.9 (21.7)	2.5 (28.7)		
Q2	12.9 (1.4)	41.2 (21.0)	51.2 (31.0)	10.0 (18.6)		
Q3	18.1 (2.5)	57.4 (59.6)	86.0 (81.7)	28.5 (27.9)		
Q4	30.1 (6.4)	48.9 (62.0)	80.8 (96.6)	31.9 (42.1)		
Single-parent households (%)					**0.0171**	0.0935
Q1	4.2 (1.7)	26.4 (23.7)	34.8 (27.8)	8.4 (21.5)		
Q2	8.1 (1.1)	40.5 (47.5)	50.4 (64.0)	9.9 (24.0)		
Q3	12.7 (1.8)	30.5 (22.8)	49.2 (29.5)	18.7 (20.2)		
Q4	20.8 (5.9)	74.7 (60.3)	111.8 (94.6)	37.1 (42.5)		
Minority (%)					0.1213	0.0380
Q1	31.9 (11.8)	25.7 (23.8)	34.3 (28.0)	8.6 (31.3)		
Q2	65.0 (9.6)	26.6 (18.3)	38.0 (15.3)	11.4 (17.1)		
Q3	91.2 (4.0)	46.8 (47.9)	74.9 (73.9)	28.2 (32.3)		
Q4	98.5 (0.8)	76.1 (61.4)	104.0 (96.0)	27.9 (42.0)		
Speaks English less than well (%)						
Q1	0.4 (0.3)	66.0 (67.2)	95.5 (99.4)	29.5 (44.2)	0.3945	0.0003
Q2	1.7 (0.9)	59.8 (48.1)	74.5 (76.7)	14.7 (39.6)		
Q3	8.0 (2.2)	26.2 (14.7)	38.5 (26.1)	12.3 (24.0)		
Q4	17.0 (5.4)	28.4 (26.8)	48.9 (40.0)	20.5 (19.9)		
Uninsured (%)						
Q1	5.0 (2.0)	34.3 (26.9)	40.1 (22.2)	5.9 (28.8)	0.1672	0.0290
Q2	8.6 (0.4)	49.8 (50.0)	75.2 (67.6)	25.4 (24.4)		
Q3	11.0 (1.2)	59.3 (66.2)	86.6 (106.6)	27.2 (44.8)		
Q4	17.0 (2.9)	35.8 (32.0)	53.1 (42.3)	16.3 (25.4)		

*Note:* Boldface indicates statistical significance (*p*<0.05).

Adj, adjusted; Q, quartile.

## DISCUSSION

OODs in Chicago increased by 45% from 2019 to 2020. Chicago Community Areas with the highest degree of social vulnerability before the pandemic already had a 2.8 times higher rate of OODs than those in the least vulnerable communities. The increase in OOD rate observed from 2019 to 2020 was 10.2 times higher in the most vulnerable communities than in the least vulnerable communities.

Although COVID-19 has seized the attention of policymakers and the public, the epidemic of addiction and overdose that preceded it remains unabated and appears to have been exacerbated owing to COVID-19 in Chicago. Although identifying causal links was outside the scope of this study, we conceptualize that COVID-19 has further exacerbated socioeconomic disparities, which in turn has intensified the opioid crisis. This finding is not unexpected because evidence indicates that low-income populations are more vulnerable at all stages of a catastrophic event, as are racial and ethnic minorities, children, older adults, and people living with disabilities.^16^

### Limitations

This study also highlights limitations when applying the Centers for Disease Control and Prevention SVI to OODs. First, although most OODs in Chicago were among the Black/African American community, the minority social vulnerability indicator did not reach statistical significance. The term minority includes all people of color with a wide variety of backgrounds and experiences. However, it does not account for the fact that some racial/ethnic minority groups, particularly the Latinx and Asian communities, have far lower rates of OODs than the Black/African American community, thus attenuating the true effect. Simply grouping individuals as White versus non-White oversimplifies the complex relationship ancestry, appearance, biology, and culture play into an individual's lived experience and overall health outcomes.^17^ With the Latinx and Asian communities being the fast-growing population in the U.S., the relative proportion of Blacks within the minority status category will be diluted, thus disguising the disparities observed within this racial minority group.^18^

Second, at the individual level, unemployment has generally been associated with increased rates of opioid overdose. However, the unemployment indicator did not show a statistical association in this study. The term unemployed includes only those eligible to participate in the labor market and actively looking for employment.^19^ Although Black men have the highest unemployment rates of any race/sex group, this rate is still likely underestimated owing to an undercount of such men in surveys and the large population of Black men incarcerated at any time.^20^ More recent national estimates, which include the incarcerated population, indicate that among working-age Black men, over a third are not working, and over a quarter have not worked in the past year.^21^ Those incarcerated accounted for a third of those out of the labor force; adjusting for those undercounted, which is estimated to be around 20% of Black men, only further widens this disparity.^21^ This has important implications for this study because Black males account for most OODs in Chicago, and thus the neighborhoods in which they reside are likely undercounting the true unemployment rate. The use of an alternative measure such as employment-to-population ratio would help to account for those that are unemployed but not in the workforce and likely make the measurement 3–4 times worse than currently estimated by the unemployment rate alone.^22^

Neighborhoods represent significant spatial locations where culture is shared, social interaction occurs, governmental resources are allocated, and a sense of community is often seeded. Neighborhoods and the social structures contained in them can have some capacity to regulate human behavior through shared expectations that set boundaries of acceptable behavior and create cultural norms about what actions should be taken when standards are violated.^23^ This perspective then might suggest that the OODs are primarily self-contained within these lower-resourced communities and that its residents are the primary consumer of the local drug market. However, it has been estimated that approximately 30% of individuals who died of an opioid overdose had traveled 2 or more ZIP codes beyond their home ZIP code, usually to more resource-deprived and segregated neighborhoods than their home ZIP code.^24^ Thus, nonresidents are playing an important role and disrupting the neighborhood social structures. Our research shows the important role neighborhoods play in an individual's health, including OODs.

## CONCLUSIONS

Chicago Community Areas with the highest degree of social vulnerability had higher baseline and disproportionate increases in OODs than the least vulnerable Chicago Community Areas. These results highlight the urgent need for policies that better support the social and economic security of disadvantaged communities, particularly for residents who use opioids. With the number of OODs expected to surpass 1.2 million over the next decades in North America, substantial policy reform is necessary.^2^ Interventions that focus on primary prevention efforts and address social determinants of health in addition to overdose prevention and response may be more effective in supporting recovery from use disorders and further enhance the resiliency of these vulnerable communities and residents. As health system researchers, we should continue to move away from focusing only on health disparities and toward looking at root causes such as systems of structural racism. Furthermore, as healthcare professionals, we must advocate for the systematic dismantling of differences in neighborhood-level resources that stem from historic racist practices. Only by addressing underlying structures will we get closer to a day when a person's health prospects are no longer predicted by the social construct of race.^25^
